# Preoperative oral spicy stimulation for postoperative pain reduction after spinal surgery: a randomized controlled trial

**DOI:** 10.3389/fmed.2026.1825570

**Published:** 2026-05-25

**Authors:** Dan Yu, Chengkun Tao, Yifan Zhang, Kunhong Li, Wei Liang, Yongqin Chen, Bin Shu, Guangyou Duan, Yamei Zhang, He Huang

**Affiliations:** 1 Department of Anesthesiology, The Second Affiliated Hospital, Chongqing Medical University, Chongqing, China; 2 Department of Anesthesiology, Wuhan Fourth Hospital, Wuhan, Hubei, China

**Keywords:** analgesia, opioid-sparing effect, postoperative pain, spicy stimulation, spinal surgery

## Abstract

**Background:**

Oral spicy stimulation has been shown to decrease pain sensitivity; however, its efficacy for acute postoperative pain remains unclear. This study aimed to evaluate whether preoperative spicy stimulation reduces postoperative pain intensity and analgesic consumption in patients undergoing elective spinal surgery.

**Methods:**

This prospective, randomized clinical trial enrolled patients who underwent spinal surgery under general anesthesia at Wuhan Fourth Hospital, China, between November 2023 and November 2024. Patients were randomized to receive oral spicy stimulation held in the mouth for 10 min, a total of nine times preoperatively, or a placebo with identical timing. The primary outcome was the area under the curve (AUC) of rest pain scores during the first 24 h postoperatively, measured using a 0–10 numeric rating scale (NRS). Secondary outcomes included the AUC of movement pain NRS scores within 24 h, the AUC of rest and movement NRS scores within 48 h, supplemental analgesia requirements, and total analgesic consumption expressed as morphine-equivalent doses.

**Results:**

Of 106 enrolled patients, 96 were included in the final analysis. Patients receiving oral spicy stimulation had lower 24-h area under the curve (AUC) of rest pain NRS (60.0 [42.0–81.0] vs. 90.0 [64.5–120.0]; MD: –30.0 [95% CI, −45.0 to −15.0]; *p* < 0.001) and movement pain NRS (114.0 [84.0–135.0] vs. 153.0 [132.0–183.0]; MD: –39.0 [95% CI, −54.0 to −24.0]; *p* < 0.001) compared with the placebo group. Similar trends were observed for 48-h AUC of rest pain (87.0 [60.0–126.0] vs. 150.0 [108.0–186.0]; MD: −57.0 [−81.0 to −36.0]) and movement pain (210.0 [156.0–243.0] vs. 279.0 [225.0–342.0]; MD: −75.0 [−108.0 to −48.0]). Furthermore, the intervention group required fewer supplemental analgesics (31.9% vs. 53.1%; RR: 0.60 [0.37–0.98]) and consumed less total analgesic medication (116.0 ± 13.8 mg vs. 126.0 ± 14.4 mg; MD: −10.0 [−15.7 to −3.4]; *p* = 0.001). All reported *p*-values are nominal and have not been adjusted for multiple comparisons.

**Conclusion:**

Preoperative oral spicy intervention was associated with a lower acute postoperative pain burden and reduced postoperative analgesic requirements in patients undergoing spinal surgery.

**Clinical trial registration:**

Chictr.org.cn, ChiCTR2300077274.

## Introduction

Postoperative pain is a significant surgical complication that can lead to poor outcomes, disability, prolonged hospitalization, and financial burden, and remains a major challenge to the healthcare system. Recent data from the China Acute Postoperative Pain Study (CAPOPS) showed that 48.7% of patients experienced moderate to severe pain on the first postoperative day (1). Similarly, the prevalence of moderate or severe postoperative pain in the Netherlands was reported to be 41% on the first day after surgery (2). Pain severity was associated with poor recovery and lower patient satisfaction. According to the CAPOPS, the incidence of moderate-to-severe pain was 56.1% in patients who underwent spinal fusion surgery (1). Approximately 8–40% of patients who undergo lumbar spine surgery experience persistent or recurrent leg and back pain after surgery (3, 4). These findings underscore the importance of improving postoperative pain management, particularly in patients undergoing lumbar spine surgery.

Current management strategies for postoperative pain following spinal surgery rely heavily on opioids, which are associated with significant adverse effects such as nausea, vomiting, sleep disorders, and the risk of long-term dependence (5–8). Perioperative nonsteroidal anti-inflammatory drugs (NSAIDs) have demonstrated opioid-sparing effects and improved pain control after spinal surgery (9). However, NSAIDs should be used with caution in patients with renal impairment, gastrointestinal ulcers, or cardiovascular diseases, and long-term or high-dose exposure may interfere with bone healing or increase the risk of nonunion following spinal fusion (10, 11). Continuous infusion of local anesthetics can also reduce opioid requirements and early postoperative pain (12) but may increase the risk of incisional infection or local anesthetic toxicity. Therefore, effective non-pharmacological interventions are warranted to optimize pain management and reduce opioid use in patients undergoing spinal surgery. According to the guidelines of peri-operative pain management in adults, analgesic interventions should be considered across the entire peri-operative pathway, encompassing multimodal analgesia (including safe opioid use) and non-pharmacological strategies. Non-pharmacological approaches are generally low-risk, may offer analgesic benefits, and can potentially reduce both the required dosage and possible adverse effects of concomitant medications (13). In addition, patients who received preoperative pain control required fewer opioids before surgery, reported lower pain scores on the visual analog scale postoperatively, and were discharged earlier (14).

Capsaicin, the primary pungent component of chili peppers and a widely used spice worldwide, has demonstrated analgesic properties through its selective agonism of the transient receptor potential vanilloid 1 (TRPV1) receptor in several studies (15, 16). Previous study in healthy volunteers have established that brief oral stimulation with capsaicin can rapidly alter a subject’s pain sensitivity, inducing a direct analgesic effect that manifests as significantly increased pressure and cold-pain thresholds (17). Beyond localized desensitization, capsaicin-induced stimulation acts as a potent noxious trigger for the central nervous system, prompting the release of endogenous opioid peptides such as *β*-endorphin from the arcuate nucleus of the hypothalamus (18, 19). This endogenous surge can interact synergistically with exogenous opioid medications, potentially enhancing their analgesic efficacy while allowing for opioid-sparing protocols. Furthermore, the concept of Conditioned Pain Modulation (CPM)—the “pain-inhibits-pain” mechanism—provides a robust theoretical foundation for using a controlled painful stimulus to activate descending inhibitory pathways, thereby filtering subsequent nociceptive inputs (20). While single exposures have shown immediate benefits, emerging evidence from preconditioning models suggests that repeated exposure to controlled noxious stimuli can enhance the body’s internal pain-regulatory capacity (21). Consequently, we hypothesize that repeated preoperative oral chili pepper stimulation can induce a cumulative state of analgesia through both peripheral desensitization and the activation of endogenous inhibitory systems. We therefore designed this randomized controlled study to determine whether preoperative oral chili pepper stimulation can alleviate postoperative pain in patients undergoing elective spinal surgery.

## Methods

### Participants and ethics

This prospective, randomized, placebo-controlled trial was conducted in accordance with the Consolidated Standards of Reporting Trials (CONSORT) guidelines and the Helsinki Declaration. This study was approved by the Ethics Committee of Wuhan Fourth Hospital and registered at www.chictr.org.cn (ChiCTR2300077274). It was conducted at Wuhan Fourth Hospital from November 2023 to November 2024 according to predetermined inclusion and exclusion criteria. Written informed consent was obtained from all patients before trial initiation. Patients aged 20–65 years, classified as American Society of Anesthesiologists class I–III, scheduled for spinal surgery under general anesthesia, who volunteered to participate and agreed to postoperative patient-controlled intravenous analgesia (PCIA), were eligible for participation. Individuals with serious systemic organ diseases, severe gastrointestinal disorders, severe food allergies, expected postoperative mechanical ventilation, alcoholism, long-term analgesic use, or inability to participate for any reason were excluded.

### Preoperative intervention

**Figure 1:**
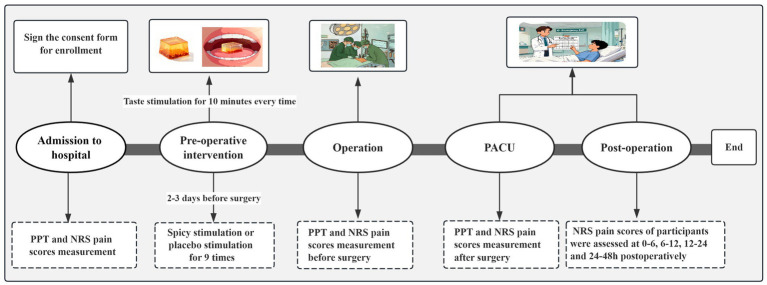
A flowchart of the trial. NRS, number rating scale; PACU, post anesthesia care unit; PPT, pressure pain threshold.

A flowchart of the trial is depicted in Figure 1. Patients were randomly assigned to either the spicy stimulation group or the placebo group. For 3 days before surgery (a total of nine consecutive interventions), participants in the spicy stimulation group were instructed to hold 18 mL (2 cm × 3 cm × 3 cm) of spicy gelatin (2% bovine skin gelatin, Sigma Aldrich, St. Louis, MO, mixed with 2 g of chili pepper powder) in the mouth for 10 min. Participants in the placebo group received 18 mL (2 cm × 3 cm × 3 cm) of tasteless gelatin prepared with pure water only. The above regimen was adapted from prior volunteer work and clinical feasibility considerations (17). All preoperative stimulations were conducted under the supervision of trained research staff, and each session was recorded in a standardized case report form. Pressure pain threshold (PPT) was measured before the spice/placebo intervention according to the protocol reported in previous studies, using a hand-held electronic mechanical algometer (YISIDA-DS2; Hong Kong, China) (22, 23).

### Randomization and blinding

Simple randomization using the sealed-envelope method was employed. A biostatistician not involved in data administration or statistical analysis generated random numbers for the two groups using SPSS. These random numbers were placed into opaque, numbered, sealed envelopes by an independent assistant. During the trial period, patients were enrolled sequentially and randomly assigned to two groups based on the envelope numbers. Interventional gelatin was prepared according to the random numbers by a researcher not involved in data collection or analysis. After the intervention, the envelopes were resealed and kept safe until study completion. Outcome evaluators and data analysts were blinded to the study protocols. The researchers who analyzed the data were not involved in treatment administration or outcome evaluation.

### Anesthesia and analgesia

After entering the operating room on the day of the procedure, the patient’s blood pressure, heart rate, electrocardiogram, and pulse oximetry were monitored regularly. An experienced anesthesiologist administered general anesthesia, and all participants received rapid-sequence induction with sufentanil 0.5–0.7 μg/kg, propofol 2–2.5 mg/kg, and cisatracurium besilate 0.15–0.2 mg/kg. Mechanical ventilation was initiated after tracheal intubation. General anesthesia was maintained with a combination of inhalation and sedation with remifentanil 0.15–0.2 μg/kg/min, sevoflurane 2%, and propofol 1–3 mg/kg/h to ensure adequate anesthetic depth. Muscle relaxants were administered as required. All spinal surgeries were performed by the same surgical team. Sufentanil 5–10 μg and flurbiprofen axetil 50 mg were administered before the end of surgery. The PCIA pumps contained 100 mL of a mixture of sufentanil (2 μg/kg), tropisetron (10 mg), and 0.9% saline. Patients were instructed to use the PCIA pump with a loading dose of 2 mL, a background infusion rate of 2.0 mL/h, a self-controlled dose of 0.5 mL, and a lock-in duration of 15 min. Pain management in the PACU is the responsibility of the anesthesiology team, whereas pain management on the general ward is the responsibility of the surgical team. Rescue analgesia, administered upon patient request, consisted of a single 50 mg diclofenac sodium suppository administered rectally as the initial pharmacologic intervention; intramuscular tramadol (100 mg) was available as a secondary rescue option if analgesia remained inadequate following the initial intervention. All administration events were recorded in the electronic medical record.

### Outcomes

The primary outcome of this study was the AUC of the rest pain numeric rating scale (NRS: 0–10, with 0 indicating no pain and 10 indicating the most intense pain) during the first 24 h after surgery. Secondary outcomes included rest and movement pain NRS scores in the PACU and at 0–6 h, 6–12 h, and 24–48 h postoperatively; the AUC of movement pain during the first 24 h, and the AUC of rest and movement pain at 48 h. Arm PPT in the PACU, the incidence of rest or movement NRS ≥ 6, and PCIA press frequency within the first 48 h were also recorded. Additional analgesic requirements and total postoperative analgesia consumption were calculated using morphine-equivalent doses. Demographic characteristics (age, height, weight, ASA grade), preference for and frequency of spicy food, surgical history, admission pain NRS scores, and PPT at admission and after intervention were recorded. PPT was measured using a handheld electromechanical manometer (YISIDA-DS100, Hong Kong, China) with a 0.1-cm^2^ probe, as previously described (24, 25). Patients lay calmly on the bed while the probe was placed vertically on the brachioradialis muscle in the right forearm at three marked points spaced 1 cm apart. Measurements were taken when the patient verbalized “pain,” and the value was recorded as the PPT in kg/cm^2^. Additional outcomes included the incidence of postoperative nausea, vomiting, dizziness, abdominal distension, and uroschesis. The 24-h AUC of rest pain was the only confirmatory primary endpoint of this study. All other outcomes, including the 48-h AUC, pain scores at various time points, and secondary clinical parameters, were predefined as secondary exploratory endpoints. Given this exploratory nature, *p*-values for these secondary endpoints were not adjusted for multiple testing; therefore, they should be interpreted as hypothesis-generating and not as definitive evidence of clinical efficacy.

### Statistical analysis

For the sample size calculation, the primary endpoint was the AUC of the rest pain NRS (0–10) during the first 24 h after surgery. Based on observations from the research group’s pre-experimental data, the NRS-AUC for pain within 24 h after spinal surgery was 84.0 ± 20.9, whereas it was 70.8 ± 15.4 after spicy stimulation. Considering *α* = 0.05 (two-sided) and a power of 0.90, the required sample size for the two-arm parallel design was 84 participants (42 per group). To account for an anticipated 20% attrition rate and potential noncompliance, 106 participants (53 per group) were enrolled.

The primary efficacy analysis was conducted on a per-protocol (PP) population, which included all randomized participants who completed the study and provided primary outcome data. Participants who withdrew from the study prior to the primary endpoint assessment were excluded from the analysis.

Statistical analyses were performed using IBM SPSS Statistics for Windows, version 26.0. Statistical significance was defined as a two-tailed *p*-value < 0.05. Data are expressed as the mean ± SD, number (frequency), or median (interquartile range), depending on data characteristics. The AUC of pain scores at 24 h postoperatively, the primary outcome, was compared between groups using independent-samples t-tests or Mann–Whitney U tests, as appropriate. Mean differences (MD) with 95% confidence intervals (CI) were calculated. Other preoperative and intraoperative variables, including age, weight, height, and postoperative outcomes such as total opioid consumption, were compared using independent-samples t-tests for normally distributed data and Mann–Whitney U tests for non-normally distributed variables. Categorical data, including sex and ASA grade, were analyzed using the chi-squared test or Fisher’s exact test.

To determine the influence of baseline and intraoperative variables (sex, age, BMI, ASA class, preference and frequency of spicy foods, surgical history, preoperative pain score, PPT, operative time, and preoperative analgesic use) and the grouping factor (spicy stimulation vs. placebo) on the AUC of rest pain NRS within 24 h after surgery, univariate linear regression analyses were first performed. Variables with *p* < 0.1 in the univariate analyses were entered into a multivariable linear regression model to assess independent factors associated with the AUC of rest pain NRS. Model assumptions, including linearity, normality of residuals, and absence of multicollinearity (variance inflation factors < 5), were verified for all regression analyses.

## Results

**Figure 2:**
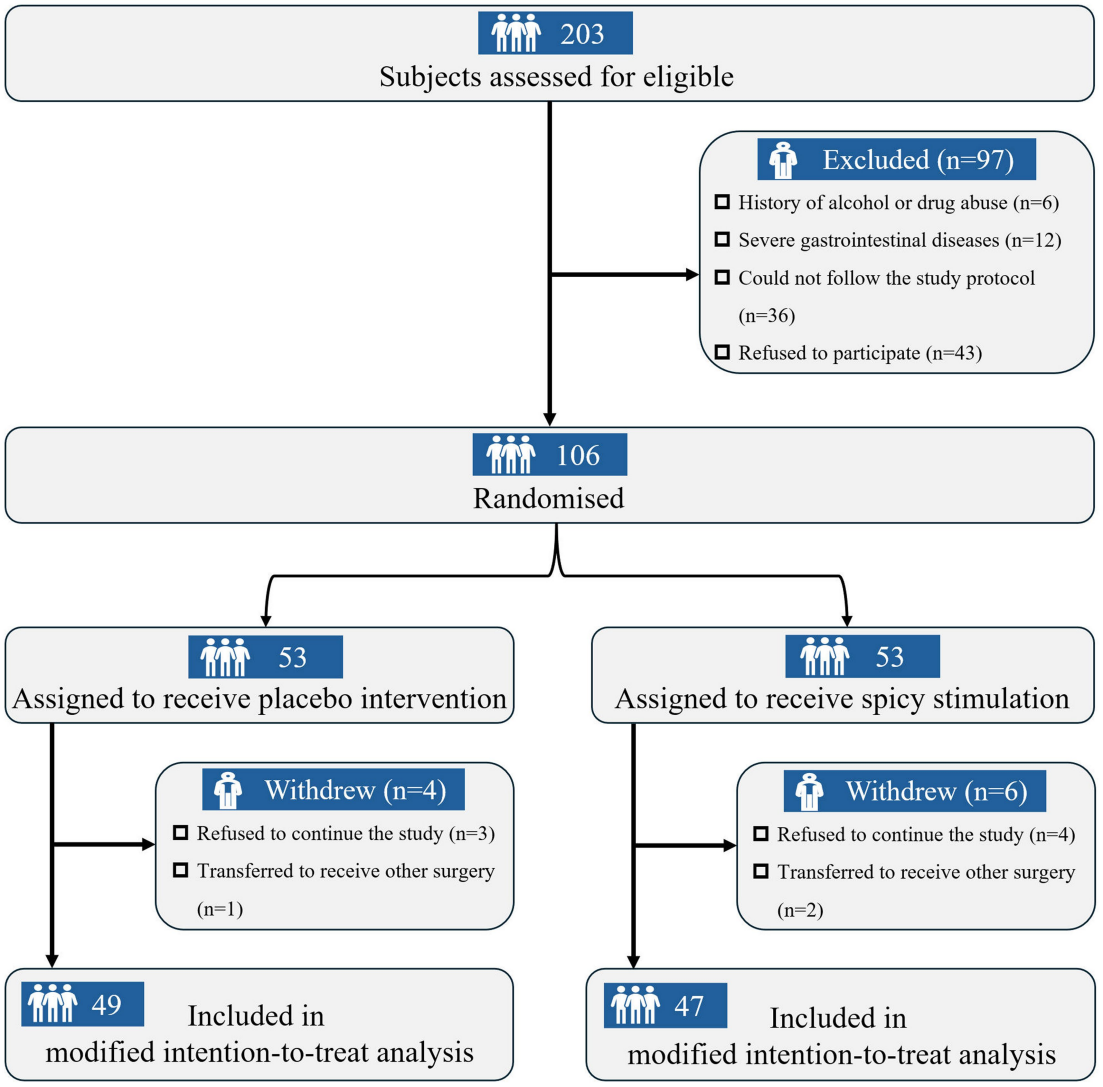
Research flow chart.

Among the 203 recruited patients, 97 were excluded for various reasons, including a history of alcohol or drug abuse, severe gastrointestinal disease, and refusal to participate. A total of 106 patients were randomly assigned to one of the two intervention groups. Ten patients (four from the placebo group and six from the spicy intervention group) withdrew from the trial during the study period because they either refused to continue or opted for alternative surgical procedures. Ultimately, 96 patients were included in the final per-protocol analysis (Figure 2). The demographic characteristics and baseline data of the included patients are shown in Table 1. No significant differences were observed between the groups.

**Table 1:** Demographic and baseline data of the included patients in spicy stimulation and placebo groups.

Variables	Placebo group (*n* = 49)	Spicy stimulation group (*n* = 47)
Age, year	53.4 ± 8.9	54.2 ± 8.9
Age group		
Age ≥ 60	13 (26.5%)	17 (36.2%)
Age < 60	36 (73.5%)	30 (63.8%)
Sex		
Male	23 (46.9%)	22 (46.8%)
Female	26 (53.1%)	25 (53.2%)
Height, cm	166.9 ± 7.1	163.4 ± 7.0
Weight, kg	68.2 ± 10.6	66.0 ± 10.5
BMI, kg/m^2^	24.4 ± 2.5	24.6 ± 3.1
BMI group		
BMI ≥ 28	21 (42.9%)	17 (36.2%)
BMI < 28	28 (57.1%)	30 (63.8%)
ASA grade		
Classification II	36 (73.5%)	27 (57.4%)
Classification III	13 (26.5%)	20 (42.6%)
NRS of preference for spicy food	5.0 (3.0–6.0)	5.0 (3.0–6.0)
NRS of frequency for spicy food	6.0 (5.0–10.0)	6.0 (4.0–10.0)
History of surgery		
Yes	26 (53.1%)	22 (46.8%)
No	23 (46.9%)	25 (53.2%)
Pain NRS at admission	6.4 ± 2.2	6.0 ± 2.1
Pain NRS at admission≥6		
Yes	26 (53.1%)	20 (42.6%)
No	23 (46.9%)	27 (57.4%)
Pressure pain threshold, kg/cm^2^	8.0 ± 1.6	7.9 ± 1.7

Data are presented as mean ± standard deviation, number (percentage), or median (interquartile range). BMI, Body mass index; ASA, American Society of Anesthesiologists; NRS, number rating scale.

Preoperative and intraoperative data are summarized in Table 2. During the preoperative period, the spicy intervention group exhibited lower pain scores compared with the placebo group (5.0 [3.0–6.0] vs. 5.0 [4.0–7.0]; MD: −1.0 [−2.0 to 0.0]; nominal *p* = 0.036). The incidence of pain scores ≥ 6 was also lower in the intervention group (17.0% vs. 36.7%; RR: 0.46 [0.22–0.96]; nominal *p* = 0.03). No notable differences were observed between groups in arm pressure pain threshold, intraoperative analgesic use, or duration of surgery.

**Table 2:** Preoperative and intraoperative data of the included patients receiving spicy stimulation and placebo intervention.

Variables	Placebo group (*n* = 49)	Spicy stimulation group (*n* = 47)	Estimated effects (95% CI)	*p* values
Pain NRS before surgery	5.0 (4.0–7.0)	5.0 (3.0–6.0)	Mean difference: −1.0 (−1.9 to −0.1)	0.036
Pain NRS before surgery≥6	18 (36.7%)	8 (17.0%)	Relative risk: 0.46 (0.22 to 0.96)	0.030
Pressure pain threshold before surgery, kg/cm^2^	8.0 ± 1.7	7.9 ± 1.7	Mean difference: 0.0 (−0.1 to 0.1)	0.901
Preoperative analgesic using	8 (16.3%)	9 (19.1%)	Relative risk: 1.17 (0.49 to 2.78)	0.717
Surgery duration, hour	2.0 (1.5–2.6)	2.0 (1.5–3.0)	Mean difference: 0.1 (−0.5 to 0.3)	0.541
Surgery duration≥2 h	26 (53.1%)	30 (63.8%)	Relative risk: 1.20 (0.86 to 1.69)	0.285
Sufentanil consumption, μg	43.9 ± 4.7	43.2 ± 5.6	Mean difference: −0.8 (−1.4 to 2.9)	0.469
Remifentanil consumption, mg	1.2 (0.9–1.8)	1.2 (0.9–1.6)	Mean difference: 0.0 (−0.2 to 0.2)	0.711

Data were presented as mean ± standard deviation, number (percentage) or median (interquartile range). NRS, number rating scale; CI, confidence interval.

**Figure 3:**
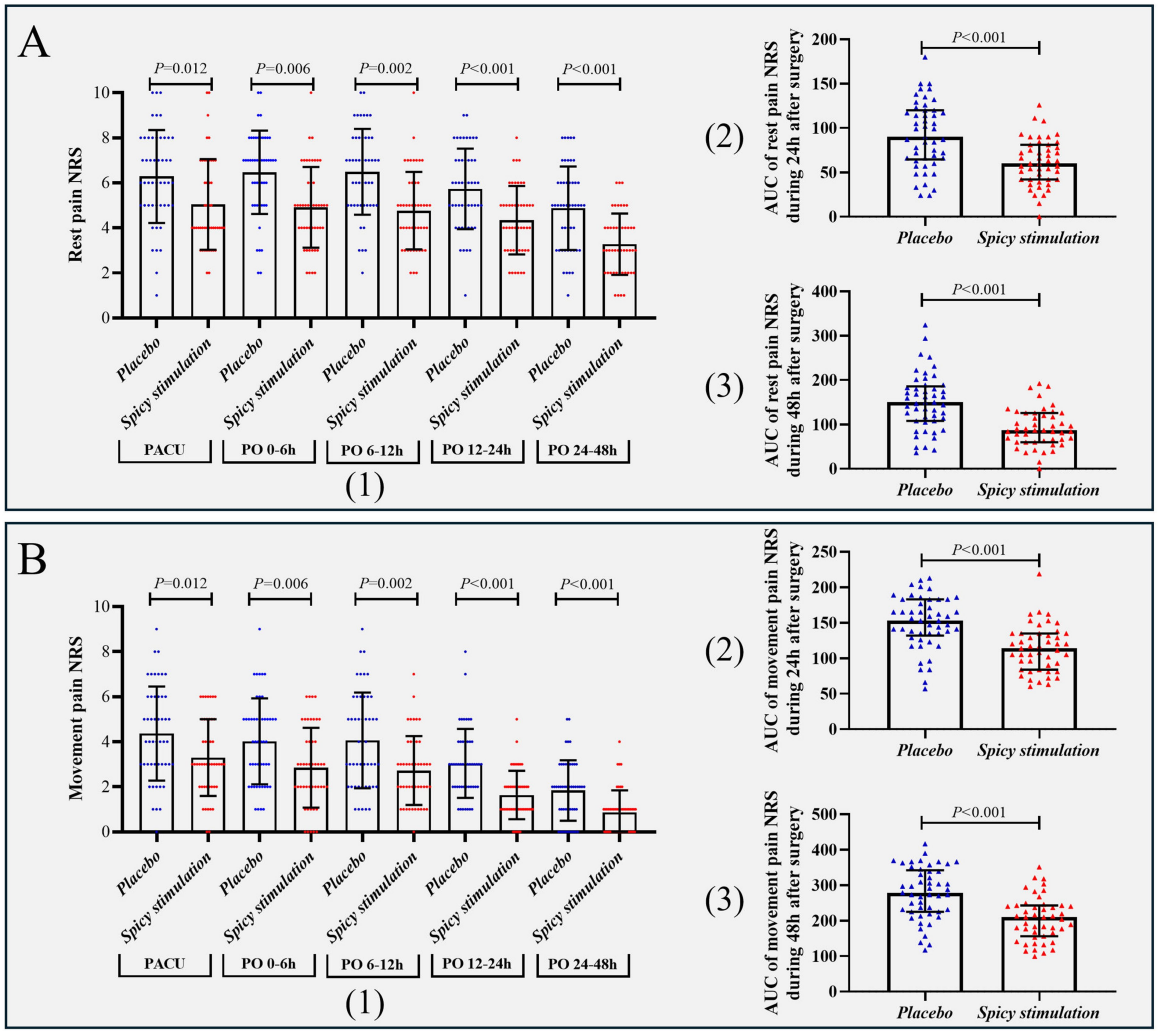
**(A)** Rest pain outcomes were compared between the intervention and placebo groups at different time points after the operation. **(B)** Movement pain outcomes were compared between the intervention and placebo groups at different time points after the operation. AUC, area under the curve. NRS, number rating scale. PACU, post anesthesia care unit.

The 24-h AUC of rest pain, as our prespecified primary endpoint, was significantly lower in the spicy intervention group (60.0 [42.0–81.0] vs. 90.0 [64.5–120.0]; MD: −30.0 [−45.0 to −15.0]; *p* < 0.001; Figure 3A-2).

All other outcomes were considered secondary exploratory endpoints. The AUC of movement-induced pain within 24 h was lower in the spicy intervention group (nominal *p* < 0.001; Figure 3B-2). Postoperative pain scores at individual time points (Figures 3A-1,B-1) and the 48-h AUCs for both rest and movement-induced pain (Figures 3A-3, B-3) were also lower in the intervention group (all nominal *p* < 0.05). As shown in Table 3, the incidence of rest pain scores ≥ 6 within 48 h was lower in the spicy intervention group (2.1% vs. 32.7%; nominal *p* < 0.001), as was the incidence of movement-induced pain scores ≥ 6 (46.8% vs. 77.6%; nominal *p* = 0.002). Furthermore, the number of PCIA button presses (nominal *p* < 0.001), the demand for supplemental analgesia (nominal *p* = 0.036), total analgesic consumption (nominal *p* = 0.001), and the incidence of opioid-related complications were all lower in the intervention group (nominal *p* = 0.001). Specifically, abdominal distension was observed in 0/47 (0.0%) patients in the intervention group versus 9/49 (18.4%) in the control group (nominal *p* = 0.002). No notable differences were found regarding the incidence of postoperative nausea, vomiting, dizziness, or uroschesis. Given the exploratory nature of these secondary outcomes, all nominal *p*-values were not adjusted for multiple comparisons and should be interpreted with caution.

**Table 3:** Postoperative data of the included patients receiving spicy stimulation and placebo intervention during 48 h after surgery.

Variables	Placebo group (*n* = 49)	Spicy stimulation group (*n* = 47)	Estimated effects (95% CI)	*p* values
Pressure pain threshold at PACU discharge	8.5 ± 2.2	8.2 ± 2.0	Mean difference: −0.3 (−1.2 to 0.5)	0.429
Rest pain NRS ≥ 6	16 (32.7%)	1 (2.1%)	Relative risk: 0.07 (0.01 to 0.47)	<0.001
Movement pain NRS ≥ 6	38 (77.6%)	22 (46.8%)	Relative risk: 0.60 (0.43 to 0.85)	0.002
PCIA pressing frequency	2.0 (0.5–5.0)	0.0 (0.0–2.0)	Mean difference: −2.2 (−0.9 to −3.4)	<0.001
Extra analgesia requirement	26 (53.1%)	15 (31.9%)	Relative risk: 0.60 (0.37 to 0.98)	0.036
Total postoperative analgesia consumption, mg*	126.0 ± 14.4	116.0 ± 13.8	Mean difference: −10.0 (−15.7 to −4.3)	0.001
PONV	19 (38.8%)	11 (23.4%)	Relative risk: 0.60 (0.32 to 1.13)	0.104
Dizziness	2 (4.1%)	0 (0.0%)	Relative risk: NA	0.162
Abdominal distension	9 (18.4%)	0 (0.0%)	Relative risk: NA	0.002
Uroschesis	1 (2.0%)	0 (0.0%)	Relative risk: NA	0.325
Postoperative complication related opioids	28 (57.1%)	11 (23.4%)	Relative risk: 0.41 (0.23 to 0.72)	0.001

Data were presented as mean ± standard deviation, number (percentage) or median (interquartile range). CI, confidence interval; NRS, number rating scale; PACU, Post Anesthesia Care Unit; PCIA, patient controlled intravenous analgesia; PONV, postoperative nausea and vomiting.

*Calculated as equivalent dose of morphine.

All secondary outcomes are exploratory; *p*-values are nominal and not adjusted for multiple comparisons.

As shown in Table 3, within the first 48 h postoperatively, the incidence of rest pain scores ≥ 6 was lower in the spicy intervention group compared with the placebo group (2.1% vs. 32.7%; RR: 0.07 [0.01–0.47]; nominal *p* < 0.001). The incidence of movement-induced pain scores ≥ 6 was also lower in the intervention group (46.8% vs. 77.6%; RR: 0.60 [0.43–0.85]; nominal *p* = 0.002). The number of PCIA button presses was lower in the spicy intervention group (0.0 [0.0–2.0] vs. 2.0 [0.5–5.0]; MD: −1.0 [−2.0 to 0.0]; nominal *p* < 0.001), as was the demand for supplemental analgesia (31.9% vs. 53.1%; RR: 0.60 [0.32–1.13]; nominal *p* = 0.036). Total postoperative analgesic consumption was reduced in the spicy intervention group (116.0 ± 13.8 vs. 126.0 ± 14.4; MD: −10.0 [−15.7 to −3.4]; nominal *p* = 0.001). Furthermore, the incidence of opioid-related complications was lower in the spicy intervention group (23.4% vs. 57.1%; RR: 0.41 [0.23–0.72]; nominal *p* = 0.001). Specifically, for the key opioid-related side effect of abdominal distension, the intervention group showed a complete absence of cases (0/47, 0.0%), whereas 9 cases were reported in the control group (9/49, 18.4%; nominal *p* = 0.002). No apparent differences were found between the groups regarding the incidence of postoperative nausea, vomiting, dizziness, or uroschesis.

In the regression analysis of factors associated with the AUC of rest pain scores within 24 h postoperatively (Table 4), sex (female *vs*. male) was a significant predictor (coefficient: 19.9, 95% CI 5.9–33.9; *p =* 0.006; adjusted coefficient: 19.8, 95% CI 7.1–32.4; *p =* 0.003). Detailed pain outcome comparisons between the two treatment groups, stratified by sex, are provided in Supplementary Tables S1, S2. Other factors, including age, BMI, ASA classification, preference and frequency of spicy food, surgical history, preoperative pain scores, and pain thresholds, showed no significant associations. Notably, the comparison between the intervention groups revealed that the spicy intervention group had significantly lower AUC rest pain scores than the placebo group (regression coefficient: −30.7 [−43.9 to −17.5]; *p <* 0.001; adjusted regression coefficient: −29.2 [−42.2 to −16.3]; *p <* 0.001), indicating that spicy intervention provided a substantial postoperative pain management benefit.

**Table 4:** Effects of baseline factors and intervention group on area under the curve of rest pain NRS during 24 h after surgery.

Variables	Regression coefficient (95%CI)	*p* values	Adjusted regression coefficient (95%CI)	*p* values
Sex (female *vs.* male)	19.9 (5.9 to 33.9)	0.006	19.8 (7.1 to 32.4)	0.003
Age, year	1.1 (−0.7 to 0.9)	0.785		
BMI, kg/m^2^	0.0 (−2.6 to 2.6)	0.999		
ASA classification (III *vs.* II)	4.6 (−10.8 to 19.9)	0.555		
NRS of preference for spicy food	0.2 (−2.8 to 3.1)	0.910		
NRS of frequency for spicy food	−0.7 (−3.2 to 1.8)	0.595		
History of surgery (yes *vs.* no)	3.7 (−3.9 to 11.3)	0.333		
Pain NRS at admission	1.2 (−2.2 to 4.6)	0.500		
Pressure pain threshold at admission, kg/cm^2^	23.3 (−21.3 to 67.9)	0.303		
Preoperative analgesic using (yes *vs.* no)	2.7 (−16.4 to 21.8)	0.778		
Pain NRS before surgery	3.1 (−0.1 to 6.2)	0.060	1.4 (−1.4 to 4.3)	0.277
Pressure pain threshold before surgery, kg/cm^2^	22.3 (−20.0 to 64.7)	0.298		
Surgery duration, hour	0.2 (−8.3 to 8.6)	0.969		
Intraoperative remifentanil using, mg	1.9 (−12.3 to 16.0)	0.262		
Intraoperative sufentanil using, μg	−0.9 (−2.3 to 0.5)	0.217		
Group (spicy stimulation *vs.* placebo)	−30.7 (−43.9 to −17.5)	<0.001	−29.2 (−42.2 to −16.3)	<0.001

BMI, Body mass index; ASA, American Society of Anesthesiologists; NRS, number rating scale; CI, Confidence interval.

## Discussion

This study demonstrated that preoperative intervention with oral spicy stimulation improved postoperative pain control in patients undergoing elective spinal surgery. Postoperative pain NRS scores at multiple time points were consistently lower in the spicy intervention group compared with the placebo group. In addition, participants who received oral spicy stimulation required fewer additional analgesic interventions, consumed lower doses of analgesics, and experienced a reduced incidence of opioid-related complications compared with participants in the control group. These secondary findings should be considered exploratory because *p* values were not adjusted for multiple comparisons and therefore require confirmation in future large-scale trials.

Beyond statistical significance, the clinical relevance of our findings merits careful consideration. While a universally accepted Minimal Clinically Important Difference (MCID) for the 24-h AUC of rest pain remains to be established, the observed treatment effect in our study is supported by a consistent pattern across multiple clinically oriented endpoints. First, the intervention resulted in a sustained reduction in pain scores, with differences of approximately 1–2 points on the Numerical Rating Scale (NRS) at several postoperative intervals, a threshold widely recognized as clinically meaningful in acute postoperative pain management. Second, this reduction translated into objective clinical benefits, including a lower incidence of severe pain (NRS ≥ 6), reduced demands for supplemental analgesia, fewer PCIA button presses, and lower total opioid consumption. Collectively, these convergent outcomes suggest that the observed benefit is likely to be clinically meaningful rather than statistically significant alone.

However, we acknowledge that the interpretation of these findings must account for potential baseline imbalances. We observed that patients in the spicy stimulation group reported lower pain scores immediately prior to surgery compared with the control group (*p* = 0.036). To determine whether this baseline difference accounted for the improved postoperative pain trajectories, we included preoperative pain as a covariate in our multivariable regression model. The analysis revealed that preoperative pain was not independently associated with the 24-h AUC of rest pain (*p* = 0.277), whereas the intervention effect remained robust and statistically significant (*p* < 0.001). These results suggest that the treatment effect is unlikely to be driven by the observed baseline imbalance. Beyond the statistical robustness of our findings, the observed analgesic efficacy aligns with broader physiological evidence. Our findings are consistent with previous studies indicating that oral spicy stimulation can induce sustained analgesia even after the initial sensation has subsided (17). This analgesic effect likely arises from multiple complementary mechanisms beyond immediate taste perception. One plausible explanation is a preemptive analgesic effect. Administered before surgical injury, spicy stimulation may modulate nociceptive processing in advance, thereby improving postoperative pain trajectories and reducing subsequent analgesic requirements. This interpretation is consistent with prior evidence supporting preemptive analgesia in spinal and orthopedic surgery (26–28). Second, capsaicin-containing stimuli activate Transient Receptor Potential Vanilloid 1 (TRPV1) channels and may initially elicit a nociceptive response (29). As a form of nociceptive input, oral spicy stimulation may engage endogenous inhibitory pathways, including conditioned pain modulation (CPM). Sustained exposure or higher doses can induce peripheral desensitization and reduce neuropeptide release, thereby decreasing afferent input to the central nervous system (30, 31), which may contribute to postoperative analgesia. Additional evidence suggests that the endogenous opioid system may contribute to the analgesic effect of capsaicin. Subcutaneous administration of capsaicin has been shown to elevate *β*-endorphin levels in the cerebrospinal fluid (CSF), which subsequently act on *μ*, *δ*, and *κ* opioid receptors (18, 19). Moreover, capsaicin may promote the transport of β-endorphin into the ventriculocisternal CSF, thereby producing central analgesia (32). Taken together, these observations suggest that the analgesic effect of oral spicy stimulation may involve a combination of preemptive modulation of nociception, TRPV1-related desensitization and central pain modulation, and possible engagement of the endogenous opioid system.

Notably, sex was identified as an independent predictor of postoperative pain intensity in this study, with female patients exhibiting significantly higher pain intensity compared with male patients. However, the study was neither prospectively designed nor statistically powered to evaluate sex-by-treatment interactions, and subgroup sample sizes were insufficient to support robust inference. Consequently, while our findings confirm sex as an independent correlate of postoperative pain, they do not establish sex-specific efficacy of spicy stimulation. Definitive assessment of potential sex differences in the analgesic response to spicy stimulation requires future randomized controlled trials with adequate statistical power and pre-specified sex-stratified analyses. Furthermore, in our cohort, preoperative spicy stimulation did not affect the PPT. This contrasts with a healthy volunteer study (17), which reported increases in PPT following capsaicin-based spicy stimulation, an effect absent in our surgical patients. Compared with healthy individuals, patients with spinal disorders typically exhibit pre-existing chronic pain, dysfunctional nociceptive processing, and established central sensitization prior to surgical intervention. Their baseline pain sensitivity is consequently elevated due to sustained nociceptive input, maladaptive spinal cord plasticity, and compensatory adaptations within endogenous pain modulation systems. These interrelated pathophysiological alterations may attenuate or obscure capsaicin-induced changes in PPT—effects that are consistently detectable in healthy volunteers characterized by normative nociceptive function and absence of pre-existing sensitization (33, 34). In addition, multimodal perioperative analgesic regimens may have attenuated measurable PPT differences. Nevertheless, preoperative oral spicy stimulation offers a low-cost, easily administered, non-pharmacological adjunct to multimodal perioperative analgesic regimens in spinal surgery. Our findings suggest that this intervention may reduce postoperative pain burden and opioid requirements during the early postoperative period. Given the well-documented risks of opioid exposure, this opioid-sparing effect may have meaningful clinical value. In our cohort, reduced opioid consumption was accompanied by fewer opioid-related adverse events, including abdominal distension.

Several limitations should be considered. First, complete participant blinding was challenging because the capsaicin-containing gelatin had distinct sensory characteristics compared with the control gelatin. This may have influenced subjective pain reporting and introduced expectancy-related bias. Second, despite our multivariable adjustment, a baseline imbalance in preoperative pain scores was observed between groups. Although our adjusted analysis demonstrated that this imbalance did not independently influence the primary outcome, the possibility of residual confounding due to the modest sample size cannot be entirely ruled out. Third, our primary efficacy analysis was based on a per-protocol population rather than a strict intention-to-treat approach. While the 9.4% withdrawal rate was low and balanced between the control (*n* = 4) and spicy stimulation (*n* = 6) groups, and the reasons for withdrawal (e.g., refusal to continue, change in surgical plan) were generally unrelated to the intervention, this could theoretically introduce attrition bias. Future trials should employ rigorous intention-to-treat principles to further validate these findings. Fourth, the optimal dosing parameters (e.g., concentration, timing, and frequency) for maximal benefit have not yet been established. Fifth, our sample was restricted to patients who underwent spinal surgery; thus, generalizability to other surgical populations remains untested. Finally, the present study did not include direct physiological validation, such as measurement of *β*-endorphin levels in plasma or cerebrospinal fluid. In addition, long-term outcomes, including chronic postoperative pain and functional recovery, were not assessed. Future multicenter trials should address these gaps by optimizing dose and timing, ensuring safety across diverse populations, and evaluating long-term efficacy. Additionally, such trials should explore integrating spicy stimulation with regional anesthesia and non-opioid analgesics within a comprehensive multimodal analgesic regimen.

## Conclusion

In this single-center randomized trial, preoperative oral spicy stimulation was associated with a lower acute postoperative pain burden and reduced postoperative analgesic requirements after spinal surgery. As a low-cost, easy-to-administer, non-pharmacological intervention, it is a promising adjunct to perioperative pain management, with the notable advantage of potentially reducing opioid use—addressing a key postoperative care challenge. These findings require further validation. Specifically, future research should focus on larger RCTs to confirm generalizability, mechanistic studies on underlying pathways, and longitudinal studies to evaluate durability and optimize parameters. Such work will aid clinical translation.

## Data Availability

The original contributions presented in the study are included in the article/Supplementary material, further inquiries can be directed to the corresponding authors.
