# Supplementary material for: Preoperative oral spicy stimulation for postoperative pain reduction after spinal surgery: a randomized controlled trial
Source: Front Med (Lausanne). 2026 May 25;13:1825570. doi: 10.3389/fmed.2026.1825570 (PMC13243222; doi:10.3389/fmed.2026.1825570)
Supplement: Supplementary file 1 [file Table_1.DOCX]

Table S1 Comparison of Pain Outcomes Between Placebo and Spicy Stimulation Group in Male Subgroup

| Variables | Placebo group **(n=23)** | Spicy stimulation group **(n=22)** | Location Shift **(95%CI)** | *P* values |
| --- | --- | --- | --- | --- |
| Rest pain NRS at PACU | 4.00 [3.00, 6.00] | 3.00 [2.00, 3.75] | -1.00 (-2.00, 0.00) | **0.025** |
| Movement pain NRS at PACU | 6.00 [5.00, 7.00] | 4.00 [4.00, 5.75] | -1.00 (-3.00, 0.00) | **0.025** |
| Rest pain NRS at 0-6h postoperatively | 4.00 [2.00, 5.00] | 2.50 [2.00, 4.75] | -1.00 (-2.00, 0.00) | 0.101 |
| Movement pain NRS at 0-6h postoperatively | 7.00 [5.00, 7.00] | 5.00 [4.00, 5.00] | -2.00 (-3.00, 0.00) | **0.009** |
| Rest pain NRS 6-12h postoperatively | 3.00 [2.00, 5.00] | 2.00 [1.25, 3.00] | -1.00 (-2.00, 0.00) | 0.069 |
| Movement pain NRS 6-12h postoperatively | 5.00 [5.00, 7.00] | 4.00 [3.00, 5.00] | -2.00 (-3.00, -1.00) | **0.005** |
| Rest pain NRS 12-24h postoperatively | 2.00 [2.00, 3.00] | 1.00 [1.00, 2.00] | -1.00 (-2.00, -1.00) | **<0.001** |
| Movement pain NRS 12-24h postoperatively | 5.00 [4.00, 6.00] | 4.00 [3.00, 5.00] | -1.00 (-2.00, 0.00) | 0.052 |
| Rest pain NRS 24-48h postoperatively | 1.00 [0.50, 2.00] | 0.50 [0.00, 1.00] | -1.00 (-1.00, 0.00) | **0.024** |
| Movement pain NRS 24-48h postoperatively | 4.00 [3.00, 5.00] | 3.00 [2.00, 4.00] | -1.00 (-2.00, 0.00) | **0.031** |
| AUC of rest pain NRS during 24h after surgery | 75.00 [57.00, 106.50] | 55.50 [37.50, 74.25] | -27.00 (-45.00, -6.00) | **0.011** |
| AUC of movement pain NRS during 24h after surgery | 138.00 [120.00, 160.50] | 105.00 [85.50, 122.25] | -33.00 (-54.00, -13.50) | **0.002** |
| AUC of rest pain NRS during 48h after surgery | 126.00 [93.00, 156.00] | 75.00 [55.50, 101.25] | -48.00 (-76.54, -18.00) | **0.002** |
| AUC of movement pain NRS during 48h after surgery | 231.00 [199.50, 295.50] | 183.00 [144.75, 229.50] | -57.00 (-93.04, -18.00) | **0.009** |

*All differences were calculated as "Spicy stimulation group - Placebo group", with negative values indicating lower pain intensity or smaller area under the curve (AUC) in the spicy stimulation group relative to the placebo group. Abbreviations: AUC ,Area Under the Curve; CI, confidence interval; NRS, number rating scale; PACU, Post Anesthesia Care Unit.*

Table S2 Comparison of Pain Outcomes Between Placebo and Spicy Stimulation Group in Female Subgroup

| **Variables** | **Placebo group (n=26)** | **Spicy stimulation group (n=25)** | **Location Shift (95%CI)** | ***P* values** |
| --- | --- | --- | --- | --- |
| Rest pain NRS at PACU | 4.50 [3.00, 6.00] | 3.00 [3.00, 5.00] | -1.00 (-2.00, 0.00) | 0.175 |
| Movement pain NRS at PACU | 6.50 [5.25, 8.00] | 5.00 [4.00, 7.00] | -2.00 (-3.00, 0.00) | **0.030** |
| Rest pain NRS at 0-6h postoperatively | 4.00 [3.00, 5.00] | 3.00 [2.00, 4.00] | -1.00 (-2.00, 0.00) | **0.022** |
| Movement pain NRS at 0-6h postoperatively | 7.00 [6.00, 8.00] | 5.00 [4.00, 7.00] | -2.00 (-3.00, -1.00) | **0.002** |
| Rest pain NRS 6-12h postoperatively | 5.00 [3.00, 6.00] | 3.00 [2.00, 4.00] | -2.00 (-3.00, -0.50) | **0.008** |
| Movement pain NRS 6-12h postoperatively | 7.00 [6.00, 8.75] | 5.00 [4.00, 6.00] | -2.00 (-3.00, -1.00) | **<0.001** |
| Rest pain NRS 12-24h postoperatively | 3.00 [2.25, 4.75] | 2.00 [1.00, 3.00] | -2.00 (-2.00, -1.00) | **<0.001** |
| Movement pain NRS 12-24h postoperatively | 6.00 [5.00, 8.00] | 5.00 [4.00, 6.00] | -2.00 (-3.00, -1.00) | **<0.001** |
| Rest pain NRS 24-48h postoperatively | 2.00 [1.25, 3.00] | 1.00 [0.00, 1.00] | -1.00 (-2.00, 0.00) | **0.002** |
| Movement pain NRS 24-48h postoperatively | 5.50 [5.00, 6.75] | 4.00 [2.00, 4.00] | -2.00 (-3.00, -1.00) | **<0.001** |
| AUC of rest pain NRS during 24h after surgery | 109.50 [73.50, 131.25] | 66.00 [51.00, 90.00] | -36.00 (-57.00, -18.00) | **0.001** |
| AUC of movement pain NRS during 24h after surgery | 165.00 [144.75, 185.25] | 120.00 [102.00, 147.00] | -45.00 (-63.00, -24.00) | **<0.001** |
| AUC of rest pain NRS during 48h after surgery | 169.50 [144.75, 203.25] | 87.00 [78.00, 126.00] | -72.00 (-96.00, -39.00) | **<0.001** |
| AUC of movement pain NRS during 48h after surgery | 306.00 [273.00, 362.25] | 213.00 [177.00, 267.00] | -87.00 (-126.00, -54.00) | **<0.001** |

*All differences were calculated as "Spicy stimulation group - Placebo group", with negative values indicating lower pain intensity or smaller area under the curve (AUC) in the spicy stimulation group relative to the placebo group. Abbreviations: AUC ,Area Under the Curve; CI, confidence interval; NRS, number rating scale; PACU, Post Anesthesia Care Unit.*
